# Security load frequency control model of interconnected power system based on deception attack

**DOI:** 10.1371/journal.pone.0298889

**Published:** 2024-02-29

**Authors:** Xin Sun, Qiuhang Tang, Qianyi Lu

**Affiliations:** 1 State Grid Zhejiang Electric Power Co., Ltd., Electric Power Science Research Institute, Hangzhou, China; 2 China Energy Engineering Group Zhejiang Electric Power Design Institute Co., LTD., Hangzhou, China; 3 State Grid Zhejiang Electric Power Co., Ltd., Wenzhou Power Supply Company, Wenzhou, China; University of the West of Scotland, UNITED KINGDOM

## Abstract

The interconnected power system connects the power grids of different regions through transmission lines, achieving power interconnection and resource sharing. However, data is transmitted through open power networks and is more susceptible to network attacks. To improve the stability of interconnected power systems under deception attacks, three scenarios of system security load frequency control were studied. Based on the construction of a dynamic model of load frequency control, an event-triggered strategy was used to reduce the communication frequency between nodes, resulting in a reduction in the amount of network transmission data. A sliding mode controller was constructed to solve the problem of event-triggered sliding mode security load frequency control. Elastic event-triggered sliding mode load frequency control for interconnected power systems under mixed attacks. The simulation results showed that using the load frequency control model triggered by events, the load frequency deviation of the interconnected power system can be stabilized at around 12 seconds, effectively saving the cost of network resources. Under the regulation of the load frequency control model based on sliding mode control, the interconnected power system stabilized in 10 seconds, reducing the load of network transmission. The elastic event-triggered sliding mode load frequency control model can ensure stable transmission of power data under various attacks and has good anti-interference performance. The results of this study have played an important role in achieving the stability of power resource supply. Compared with previous studies on individual power systems, this study solves the attack problem of interconnected power systems and considers the frequency control problem of system security loads under mixed attacks, enabling the system to recover stability faster.

## 1. Introduction

The power systems of several regions are connected using interconnection lines to obtain interconnected power systems. This system typically consists of multiple power plants, transmission networks, and distribution networks, used to supply electricity to a wide range of users [1]. The goal of an interconnected power system is to achieve efficient transmission, exchange, and electricity distribution to satisfy different regions and users [2]. Although interconnected power systems have many benefits, they are more susceptible to network attacks compared to traditional power networks. In interconnected power systems, load frequency control (LFC) and sliding mode control (SMC) are two key control strategies used to maintain stable operation and a reasonable range of frequencies [3]. In an interconnected power system, there is a certain balanced relationship between the generator and load output. When the load increases, the generator output correspondingly increases to maintain the stability of the system frequency [4]. LFC is achieved by monitoring changes in system frequency and adjusting the output of the generator to maintain the system frequency at a predetermined value [5]. By adjusting the power flow through a sliding mode controller, the node voltage in the system can be maintained stable and able to cope with system changes and fault situations. Therefore, this study constructs an event-triggered strategy-based LFC model under spoofing attacks based on event-triggered strategies, introduces a sliding mode controller, and constructs a LFC model based on SMC. Unlike previous studies, this study analyzes the problem of LFC under spoofing attacks from the perspective of interconnected power systems. In addition, the effectiveness of the method was verified from the three zone interconnected power system and the two zone interconnected power system, respectively, expanding the applicability of the research method. In terms of major contributions, the sufficient criteria for the asymptotic stability of the system are obtained through methods such as SMC with strong robustness. The research designs an elastic event-triggered strategy that has a certain delay immunity against DoS attacks. In the presence of network attacks, this strategy is beneficial for improving the utilization of limited network resources.

The study is divided into four parts. The first part discusses the research results of domestic and foreign experts and scholars on LFC and SMC. The second part constructs a LFC model based on event-triggered strategy. Based on spoofing attack conditions, a sliding mode controller is introduced to construct a LFC model based on SMC. This part considers the latency issue caused by denial-of-service (DoS) attacks, converts it into elastic event-triggered conditions, and constructs a frequency control model for interconnected power loads under mixed attacks. The third part conducts simulation experiments and analyzes the three models constructed separately. The fourth part summarizes the article and points out its shortcomings.

## 2. Related works

LFC mainly adjusts power system frequency and the exchange power of the interconnection line to achieve frequency stability. Some experts and scholars have relevant research results on LFC. S Liu et al. proposed a dynamic event-triggered mechanism based on asynchronous advantage actor critic (A3C) learning to solve the problem of communication load fluctuations in the power system. The threshold of the event-triggered function was adjusted through real-time optimization, and a new model of a decentralized LFC system was established. The results showed that the designed model effectively solved the problem of communication load [6]. J Pan et al. constructed a LFC model with time delay and uncertainty to solve the load frequency problem in the wind power integrated power system and demonstrated the stability of the system using the Lyapunov theory. The simulation results demonstrated the effectiveness and rationality of this method [7]. T Weng et al. designed corresponding control methods based on multi-agent systems under false data injection attacks to effectively control the load frequency of multi-region power systems. After verification, the proposed method was effective [8]. C N S Kalyan et al. proposed a 3DOFPID controller based on the Seagull Optimization Algorithm (SOA) to solve the LFC of a system consisting of two regions. After verification, the control effect of the proposed method was good [3].

SMC is a method for solving LFC problems and is often used in the frequency regulation of systems. Some experts and scholars have conducted relevant research on SMC. K S Sanal et al. found that the amplitude of the inverter output can be affected by load changes, and DC link voltage regulation was important in converter stability maintenance. However, a single SMC did not have any relevant controls for common regulation issues. Therefore, a coupled SMC can address the DC links voltage regulation. The simulation results showed that this scheme can achieve the stability of DC link voltage regulation in the power system [9]. T Chen et al. proposed an observer-based adaptive neural network backstepping sliding mode controller to address stability issues in power systems. To enhance robustness, SMC technology was introduced. The constructed Lyapunov function ensured the stability of the system. The effectiveness of this method was verified through examples [10]. E A A O Almatroud designed a simple sliding surface and proposed an adaptive controller with corresponding parameter update law to solve the anti-synchronization problem in two different fractional-order chaotic and hyperchaotic systems when the system parameters are uncertain. The effectiveness of the proposed anti-synchronization scheme was verified through numerical simulation [11]. R Moutchou et al. proposed first-order and second-order SMC schemes to enhance the maximum wind power of the permanent magnet synchronous generator turbine while reducing the impact of mechanical stress. The simulation results under turbulent wind speed and parameter changes showed that the proposed control method had fast identification and tracking efficiency, robustness, and significantly improved performance [12].

In summary, the research on SMC strategy in LFC is relatively mature, and it can significantly improve the stability and robustness of power systems. However, in existing research, there is relatively little involvement in the sliding mode load control problem of multi-region power systems under network attacks. Therefore, the research focuses on the interconnected power system and analyzes its security issues when subjected to network attacks, introducing an SMC strategy during this period. Compared to previous research, the research has expanded the scope of power system research, analyzed the sliding mode load control problem of multi-region power systems, and studied the security control problem under mixed network attacks.

## 3. Secure LFC model construction for interconnected power systems based on spoofing attacks

Due to frequency instability in interconnected power systems when subjected to network attacks. This chapter is divided into three parts to construct a LFC model. The first part constructs a LFC model based on an event-triggered strategy. The second part introduces a sliding mode controller based on Spoofing attack conditions and constructs a LFC model based on SMC. The third part considers the delay problem caused by DoS attacks, converts it into elastic event-triggered conditions, and constructs a frequency control model for interconnected power loads under mixed attacks.

### 3.1. LFC model construction based on event-triggered strategy

**Fig 1:**
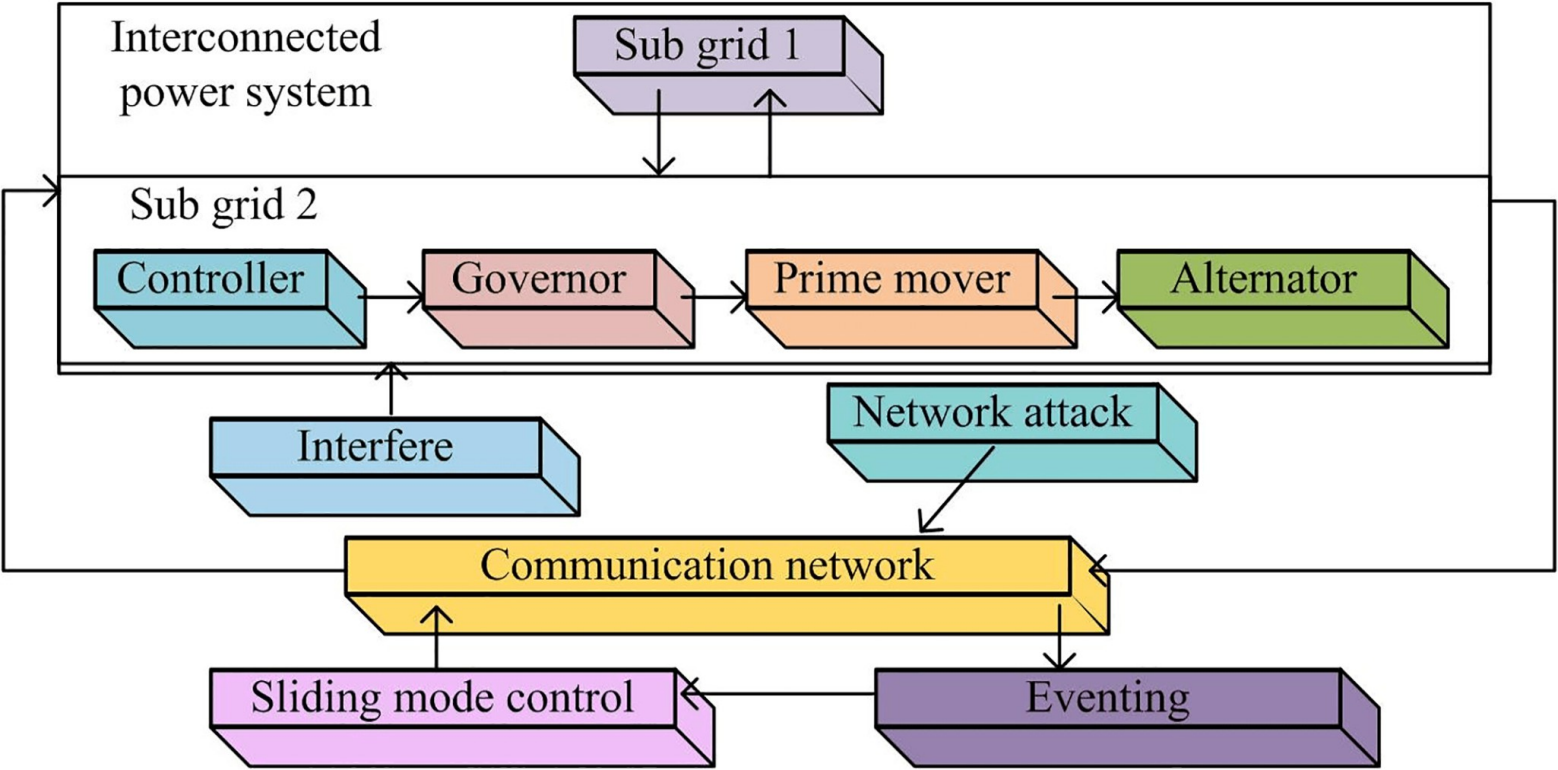
Interconnected power system control block diagram.

An interconnected power system refers to a system formed by connecting multiple power systems through transmission lines, substations, and other equipment [13]. The system can share power resources among various sub-power systems, reduce the backup capacity of the system, and improve the power supply. Frequency control can be adjusted by utilizing the interconnection of resources within the system and can also be supported by the interconnection lines in adjacent areas [14]. The control block diagram of the interconnected power system is shown in Fig 1.

In Fig 1, in an interconnected power system, the frequency variation and tie-line power of the system are kept within the allowable range under the action of a set controller. When the frequency increases, the controller can adjust the steam intake of the prime mover to reduce the active power of the generator, ensuring that the frequency of the system is within the allowable range. However, due to the interconnection between various power systems, it can easily make the network vulnerable to attacks. In contrast to DoS attacks, spoofing attacks employing randomness are particularly challenging to counter as they can evade detection, thereby heightening the complexity of network defense [15]. Moreover, deceptive attackers often constantly change their strategies and techniques to adapt to and bypass security system detection. This dynamism and adaptability require defense mechanisms to be constantly updated to cope with ever-changing threats. The dynamic model equation of LFC is shown in Eq (1).


$$\left\{\begin{array}{l} \dot{x}(t)=Ax(t)+Bu(t)+F\omega (t)\\ y(t)=Cx(t) \end{array}\right.$$ (1)

In Eq (1), *x*(*t*) denotes the system state vector, *ω*(*t*) denotes the control input, *y*(*t*) denotes the measured output signal, and *A*, *F* represent the real constant matrices with a certain dimension, *B* represents the parameter, *u*(*t*) represents the control input that has been subjected to deception attacks. In a Proportional Integral Differential (PID) controller, its input is set to the Area Control Error Signal (ACE). Based on PID control, when a deception attack occurs, the corresponding system state equation is shown in Eq (2).


$$\left\{\begin{array}{l} \dot{x}(t)=Ax(t)+G(a(t))x(t)+J(a(t))+F\omega (t)\\ y(t)=Cx(t) \end{array}\right.$$ (2)

In Eq (2), *a*(*t*) denotes the Bernoulli distribution random variable at the time of the attack, *G*, *J* represent the real constant matrices with a certain dimension. For ease of calculation, *G*(*a*(*t*)) = −(1 − *a*(*t*)) *BKC*, *J*(*a*(*t*)) = (*a*(*t*) *Bζ*(*t*) is defined, *ζ*(*t*) represents signals with limited attack energy, and *K* represents gain. When (*t*) = 1, control input *u*(*t*) = *a*(*t*)*ζ*(*t*) indicates that a spoofing attack occurred during data transmission. When (*t*) = 0, control input *u*(*t*) = −*KCx*(*t*) means that the sampling measurement has been successfully transmitted. ACE is not only related to frequency deviation [16] but also to the exchange power of the interconnection line. ACE is defined as Eq (3).


$$ACE_{i}=\beta_{i}\Delta f_{i}+\Delta P_{tie-i}$$ (3)

In Eq (3), Δ*f* represents the system frequency deviation, Δ*P*_*tie*−*i*_ represents the tie line power deviation, and *β*_*i*_ represents the conversion coefficient between power and frequency. Spoofing attacks often reduce system performance by modifying signals and sensor measurements. The damage measurement value of spoofing attacks is shown in Eq (4).


$$\bar{y}(t)=y(t)+\alpha (t)\left(-y(t)+\zeta (t)\right)$$ (4)

In traditional power systems, periodic time control schemes are often used. In the time control scheme, there is a Zero Order Hold (ZOH) [17] that enables the system to balance closed-loop performance and communication load to ensure the control performance of the controller. However, this method does not take into account the update of the input signal, making the data appear cumbersome and resulting in lower network transmission performance [18]. Therefore, the event-triggered strategy is replaced by the original control scheme. The event-triggered strategy is to compare the error of the current transmission data with the designed threshold, and only trigger at a specific time. The output from Eq (2) is substituted into the threshold conditions of traditional event-triggered strategies to obtain the triggering conditions of event-triggered strategies as shown in Eq (5) [19].


$$t_{k+1}h=t_{k}h+\underset{l\in N}{min}\{lh|e^{T}(i_{l}h)\Phi e(i_{l}h)>\sigma x^{T}(t_{k}h)\Phi x(t_{k}h)\}$$ (5)

In Eq (5), *e*(*i*_*l*_*h*) = *x*(*i*_*k*_*h*) − *x*(*i*_*l*_*h*), *i*_*k*_*h* = *t*_*k*_*h* + *lh*. *i*_*k*_*h* represents the current sampling time, *t*_*k*_*h* represents the time when the signal under triggering conditions is transmitted to the controller, *σ* represents the event-triggered parameter. *h* represents the sampling period of the load frequency. Among them, Φ = *C*^*T*^ *ρC*, *ρ* represents the trigger matrix. To introduce a time-triggered condition for each sampling instance to ascertain the transmission necessity of the collected data through the network. Since ZOH maintains the control signal unchanged until the next sampling time, it can cause excessive data. Therefore, the retention time interval Ω of ZOH is divided into a new subset in Eq (6).


$$\Omega =⋃\Omega_{l},\Omega_{l}=[i_{l}h+\tau_{t_{k}},i_{l}h+h+\tau_{t^{*}})$$ (6)

In Eq (6), $\tau_{t^{*}}$ represents the delay of all release signals *x*(*i*_*l*_*h*) reaching the actuator, as shown in Eq (7).


$$\tau_{t^{*}}\left\{\begin{array}{l} \tau_{t_{k}},l=0,1,2,\cdots ,t_{k+1}-t_{k}-2\\ \tau_{t_{k+1}},l=t_{k+1}-t_{k}-1 \end{array}\right.$$ (7)

In Eq (7), $\tau_{t_{k}}$ represents the delay within [0, *t*_*k*+1_ − *t*_*k*_ − 2], and $\tau_{t_{k+1}}$ represents the delay at *t*_*k*+1_ − *t*_*k*_ − 1. *η*(*t*) = *t* − *i*_*l*_*h*, *t* ∈ Ω_*l*_ represents the system delay, and the output feedback *u*(*t*) is represented as follows.


$$u(t)=-(1-\alpha (t))KCx(t-\eta (t))+\alpha (t)\zeta (t)+KCe(i_{l}h)$$ (8)

In the above equation, 0 ≤ *η*(*t*) ≤ *h* + *η*_*M*_, *η*_*M*_ represents the maximum allowable delay upper bound. Substituting Eq (8) into Eq (2), the time-delay model of interconnected power system LFC dependent on ACE can be obtained as shown in Eq (9).


$$\left\{\begin{array}{l} \dot{x}(t)=Ax(t)+G(\alpha (t))x(t-\eta (t))+J(\alpha (t))+BKCe(i_{l}h)+F\omega (t)\\ y(t)=Cx(t),t\in \Omega_{l} \end{array}\right.$$ (9)

*H*_∞_ is a classic control strategy often applied in networked control systems. When *ω*(*t*) = 0 and *ζ*(*t*) = 0 are satisfied, the delay model represented by Eq (8) is in a stable state. Meanwhile, under the condition $x(t)=0,t\in [-\bar{\eta },0]$ of zero initial, for any non-zero *ω*(*t*) ∈ *L*_2_[0, ∞] and *ζ*(*t*) ∈ *L*_2_[0, ∞] and a given level *γ* of *H*_∞_ anti-interference, inequality (10) can hold.


$$E\{\|y(t){\|}_{2}\}\leq \gamma E\{\|\omega (t){\|}_{2}+\|\zeta (t){\|}_{2}\}$$ (10)

Eq (10) proves that the delay model of interconnected power system LFC dependent on ACE has *H*_∞_ disturbance suppression performance.

### 3.2. LFC model construction based on sliding mode membrane control

PI controller has the characteristics of simple algorithm and convenient parameter setting and is widely used in network system control. However, PI control is often used in linear regions, ignoring nonlinear situations. At the same time, when system input undergoes significant changes, algorithms in the control system can lead to excessive control, resulting in a situation where the system runs for too long. SMC shows fast response and easy implementation, and can also solve the shortcomings of PI controllers [20]. SMC mainly involves inserting a sliding mode device at a node to regulate the power flow of the node, to maintain node voltage within a reasonable range. By adjusting the power flow through a sliding mode controller, the node voltage in the system can be maintained stable and able to cope with system changes and fault situations. SMC determines a control law by designing a set of switches to enable the system’s motion trajectory to run on a sliding surface [21]. When the model is subjected to spoofing attacks, Eq (1) is rewritten as shown in Eq (11).


$$\left\{\begin{array}{l} \dot{x}(t)=Ax(t)+Bu(t)+F\omega (t)\\ y(t)=(1-\alpha (t))Cx(t)+\alpha (t)\zeta (t) \end{array}\right.$$ (11)

**Fig 2:**
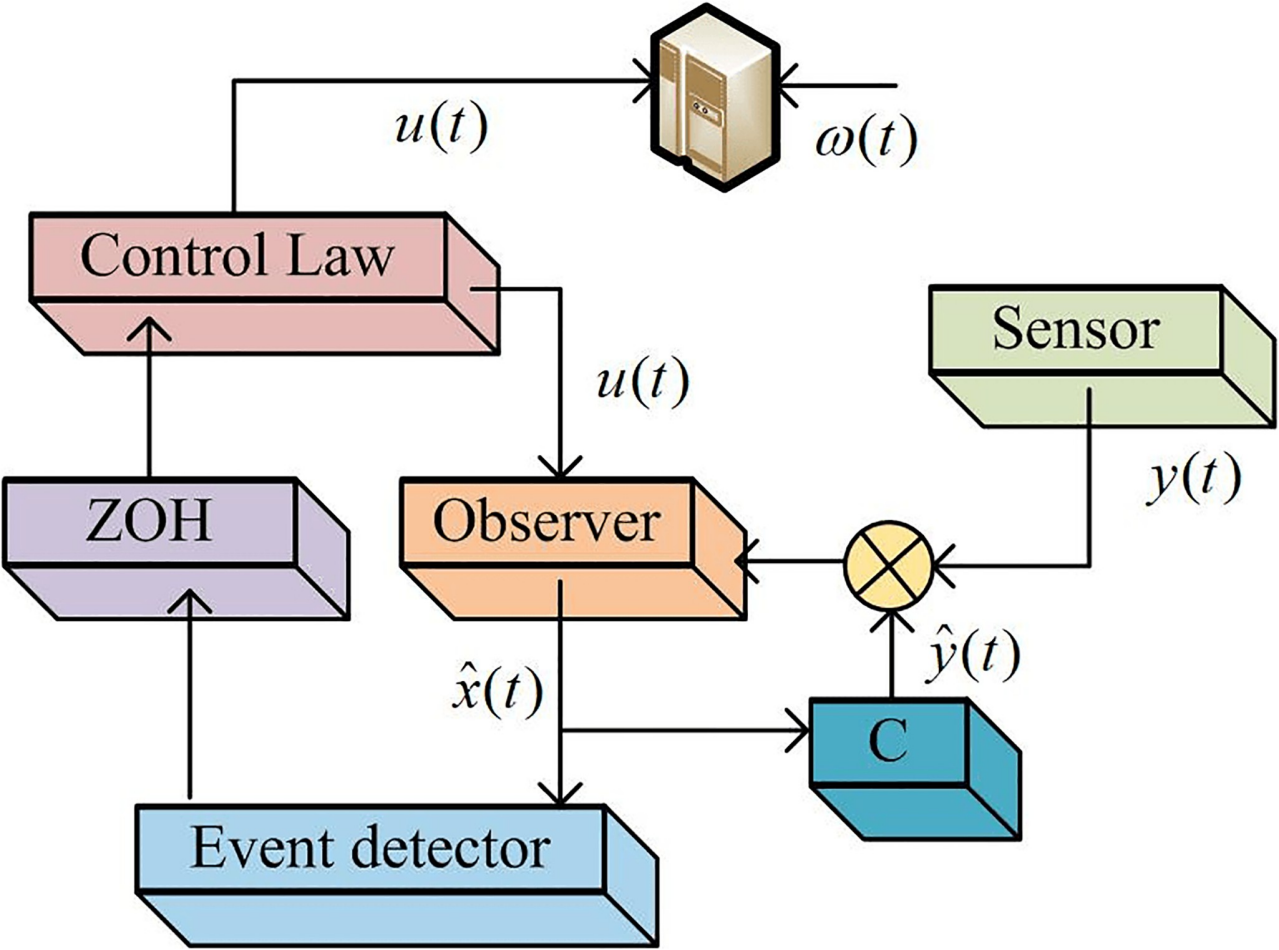
Control system based on event-triggered strategy.

In Eq (11), *ω*(*t*) represents the system input. *y*(*t*) represents sensor output. The control system using an event-triggered strategy is shown in Fig 2.

In Fig 2, an event detector is introduced to represent the channel from the Longberg observer to the SMC. Event-triggered strategies can trigger corresponding control or protection actions based on the occurrence of specific events. For example, when a serious fault or short circuit event occurs in the system, protective measures such as tripping, and isolation of circuit breakers can be triggered to safely operate equipment and systems. Therefore, the triggering conditions of the observer-based event-triggered strategy are shown in Eq (12).


$$\left\{\begin{array}{l} t_{k+1}h=t_{k}h+\underset{l\in N}{min}\{lh|e_{\hat{x}}^{T}(t)\Phi e_{\hat{x}}(t)>\sigma {\hat{x}}^{T}(t_{k}h)\Phi \hat{x}(t_{k}h)\}\\ e_{\hat{x}}(t)=\hat{x}(i_{k}h)-\hat{x}(t_{k}h) \end{array}\right.$$ (12)

In Eq (12), $\hat{x}(i_{k}h)$ represents the vector sent by ZOH to the SMC law. The expression of the Longberg observer is shown in Eq (13).


$$\left\{\begin{array}{l} \dot{\hat{x}}(t)=A\hat{x}(t)+Bu(t)+L[y(t)-\hat{y}(t)]\\ \hat{y}(t)=(1-\alpha (t))C\hat{x}(t) \end{array}\right.$$ (13)

In Eq (13), *L* represents the Longberg observer gain matrix, $\hat{x}(t)$ and $\hat{y}(t)$ represents the state and output estimation vector. Based on the observer function, the expression of the sliding mode surface is obtained in Eq (14).


$$s(t)=B^{T}X[\hat{x}(t)-\int_{0}^{t}\left(A+BK\right)\hat{x}(\theta )d\theta ]$$ (14)

In Eq (14), *K* and *X* represent the coefficient matrix. Among them, *A* + *BK* satisfies the Hurwitz matrix and *X* satisfies the non-singular matrix *B*^*T*^*XB*. Substituting Eq (14) into Eq (13) yields the expression shown in Eq (15).


$$\begin{array}{ll} \dot{s}(t) & =B^{T}X\dot{\hat{x}}(t)-B^{T}X(A+BK)\hat{x}(t)\\ & =B^{T}XBu(t)-B^{T}XBK\hat{x}(t)+B^{T}XL[(1-\alpha (t))C\varpi (t)+\alpha (t)\zeta (t)] \end{array}$$ (15)

In Eq (15), $\dot{s}(t)=0$ obtains the equivalent input as shown in Eq (16).


$$u_{eq}(t)=K\hat{x}(t)-{\left(B^{T}XB\right)}^{-1}B^{T}XL[(1-\alpha (t))C\varpi (t)+\alpha (t)\zeta (t)]$$ (16)

By defining the delay as *η*(*t*) = *t* − *i*_*k*_*h*, using Eq (12) and substituting Eq (16) into Eq (13), the dynamic equation of SMC based on event-triggered strategy is obtained in Eq (17).


$$\begin{array}{ll} \dot{\hat{x}}(t) & =A\hat{x}(t)+BK\hat{x}(t-\eta (t))-BKe_{\hat{x}}(t)+(L-B{\left(B^{T}XB\right)}^{-1}B^{T}XL)\\ & \times [(1-\alpha (t))C\varpi (t)+\alpha (t)\zeta (t)]\\ & =A\hat{x}(t)+BK\hat{x}(t-\eta (t))-BKe_{\hat{x}}(t)+\bar{B}[(1-\alpha (t))C\varpi (t)+\alpha (t)\zeta (t)] \end{array}$$ (17)

In Eq (17), $\bar{B}=L-B{\left(B^{T}XB\right)}^{-1}B^{T}XL$. $0\leq \eta (t)\leq \bar{\eta }$, $\bar{\eta }$ is a constant expressed as maximum delay upper bound. Therefore, the LFC model based on the sliding mode controller can be comprehensively obtained as shown in Eq (18).


$$\left\{\begin{array}{l} \dot{\hat{x}}(t)=A\hat{x}(t)+BK\hat{x}(t-\eta (t))-BKe_{\hat{x}}(t)+\bar{B}[(1-\alpha (t)C\varpi (t)+\alpha (t)\zeta (t)\\ \dot{\varpi }(t)=A\varpi (t)-L[(1-\alpha (t))C\varpi (t)+\alpha (t)\zeta (t)]+F\omega (t) \end{array}\right.$$ (18)

Based on the designed model, a controller can be designed as shown in Eq (19), enabling the system to converge onto the desired surface.


$$u(t)=-(\delta (t)\cdot \text{sgn}(s(t))+\tau s(t))+K\hat{x}(t)$$ (19)

In Eq (19), *τ* is a constant that is always greater than 0. sgn(⋅) represents a symbolic function. The *δ*(*t*) expression is *σ*(*t*) = ‖(*B*^*T*^*XB*)^−1^‖[‖*B*^*T*^*XLζ*(*t*)‖ + 2‖*B*^*T*^*XLCϖ*(*t*)‖], ‖⋅‖ represents the Euclidean norm.

### 3.3. Sliding mode LFC model construction triggered by elastic events in interconnected power systems under multiple attacks

**Fig 3:**
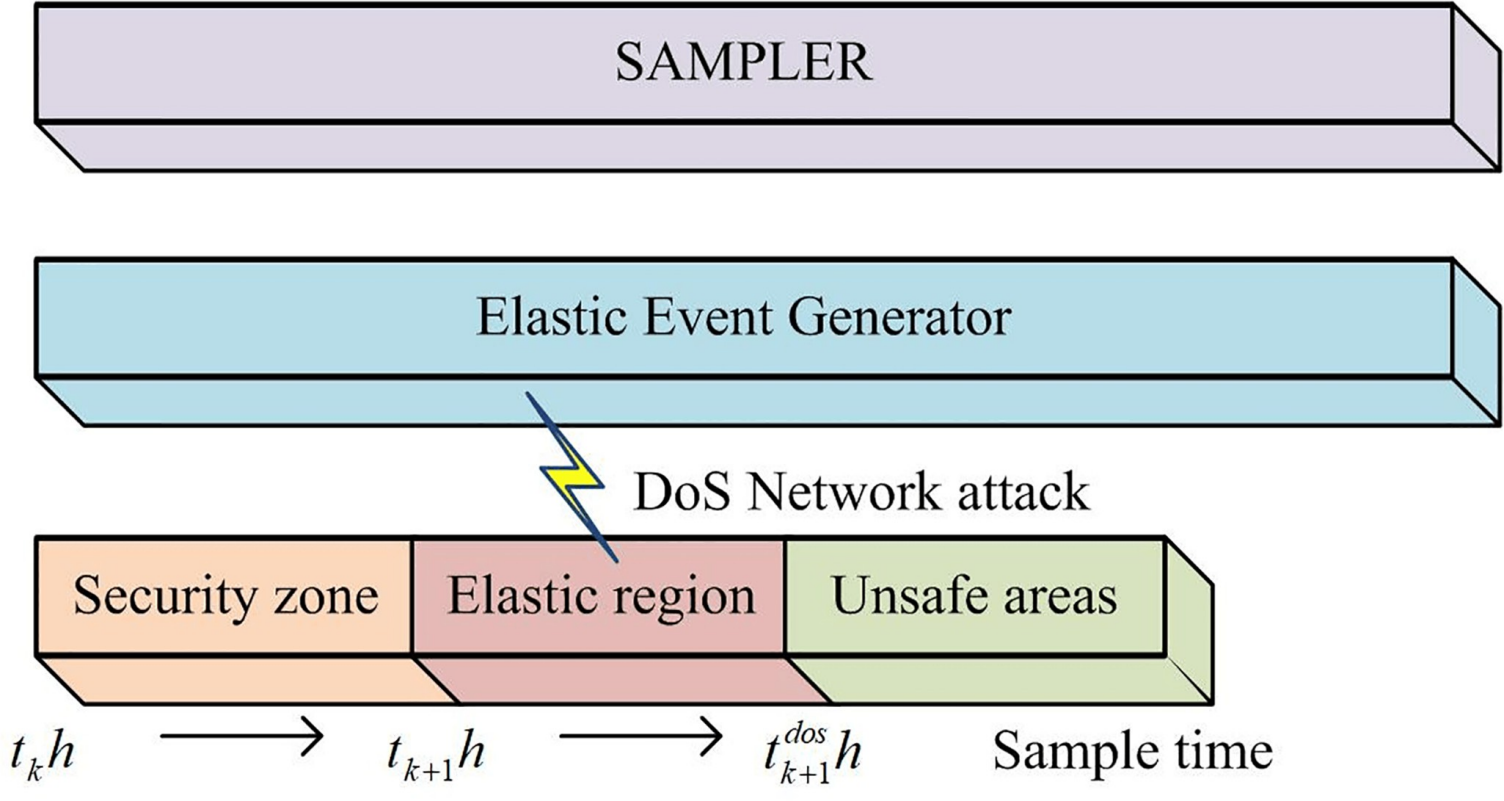
Elastic event-triggered strategy.

To solve the problem of network traffic congestion caused by limited bandwidth limitations. Existing research often involves designing an appropriate transmission strategy to address this issue. However, this solution makes network systems more susceptible to spoofing attacks and DoS attacks [22]. Thus, a security defense mechanism is established to prevent network attacks. DoS attacks can often cause network latency and affect system stability [23]. Therefore, establish a flexible event-triggered strategy to reduce unnecessary data transmission. The elastic event-triggered strategy is shown in Fig 3.

In Fig 3, elastic triggering includes three methods: the first is the safe zone, the second is the elastic zone, and the third is the unsafe zone. There is no DoS attack in the security zone, and every data can be guaranteed to be successfully transmitted. There is a DoS attack in the elastic region, which generates a small amount of latency and allows data to be transmitted. In insecure areas, there are DoS attacks and strong latency, making data unsent [24]. The event-triggered strategy filters data based on pre-set triggering conditions, as shown in Eq (20) for traditional triggering conditions.


$$t_{k+1}h=t_{k}h+\underset{l\in N}{min}\{lh|e^{T}(i_{k}h)C^{T}\rho Ce(i_{k}h)>\sigma y^{T}(t_{k}h)\delta y^{T}(t_{k}h)\delta y(t_{k}h)\}$$ (20)

In Eq (20), $e(i_{k}h)=x(i_{k}h)-x(t_{k}h),i_{k}h=t_{k}h+lh,l\in \mathbb{N},i_{k}h\in (i_{k}h,t_{k+1}h],t_{k}(k=0,1,2\cdots )$. *δ* represents a trigger matrix. *σ* represents a threshold parameter, with values between (0,1). *h* represents the load frequency sampling period and *i*_*k*_*h* is the current sampling signal. *y*(*t*) = *C*_*x*_(*t*_*k*_), *t* ∈ [*t*_*k*_*h*, *t*_*k*+1_*h*), Ψ = *C*^*T*^*ρC*. The triggering conditions under DoS attacks are shown in Eq (21).


$$t_{k+1}h=t_{k}h+\underset{l\in N}{min}\{lh|e^{T}(i_{k}h)\Psi e(i_{k}h)>\sigma x^{T}(t_{k}h)\Psi x(t_{k}h)\}$$ (21)

Due to DoS attacks and limited energy constraints in the system, *φ*(*i*_*k*_*h*) is used to represent real-time DoS attacks for each sample, as shown in Eq (22) [25].


$$\varphi (i_{k}h)=\left\{\begin{array}{l} 1DoSattack\\ 0NoDoSattack \end{array}\right.$$ (22)

Due to the limited nature of DoS attacks, finite energy DoS attacks can be described as the expression shown in Eq (23) [26].


$$\Delta_{t_{k+1}h}^{dos}=t_{k+1}^{dos}h-t_{k+1}h<\Delta_{dos}$$ (23)

In Eq (23), $t_{k+1}^{dos}h$ represents the time when the signal is transmitted to the controller during a DoS attack. Δ_*dos*_ indicates maximum DoS attack duration. From the above analysis, the system state under DoS attacks cannot be directly obtained. Therefore, a flexible time-triggering strategy based on the Longberg observer is introduced as shown in Eq (24).


$$t_{k+1}^{dos}h=t_{k}h+\underset{t}{min}\left\{t\wedge i_{k}h\left|e_{\hat{x}}^{T}(i_{k}h)\Psi e_{\hat{x}}(i_{k}h)-\varphi (i_{k}h){\left(e_{\hat{x}}^{dos}(i_{k}h)\right)}^{T}\Psi e_{\hat{x}}^{dos}(i_{k}h)>\sigma {\hat{x}}^{T}(t_{k}h)\Psi \hat{x}(t_{k}h)\right.\right\}$$ (24)

In Eq (24), $e_{\hat{x}}^{dos}(i_{k}h)$ represents the additional error on the system, and $\hat{x}(i_{k}h)$ represents the state estimation vector. By Eq (22), DoS attacks limited energy constrains triggering condition uncertainty, $\varphi (i_{k}h){\left(e_{\hat{x}}^{dos}(i_{k}h)\right)}^{T}\Psi e_{\hat{x}}^{dos}(i_{k}h)<M$. Therefore, three types of DoS attacks can be identified. The first option is that if $\varphi (i_{k}h)=0,t\in (t_{k}h,t_{k+1}^{dos}h)$, the DoS attack is ineffective, the elastic event-triggered strategy will be transformed into a regular static triggering strategy. The second type of if $\varphi (i_{k}h)=1,t\in [t_{k+1}h,t_{k+1}^{dos}h]$, the DoS attack will not affect the system’s control updates, while the third type of if $\varphi (i_{k}h)=1,t\in [t_{k+1}h,t_{k+1}^{dos}h]$, the system has been subjected to a DoS attack, resulting in a sustained delay in the attack time $\Delta_{t_{k+1}h}^{dos}$. In spoofing attacks, attackers can rearrange data in the system, causing sensors or controllers to receive false data, resulting in the system not functioning properly [27]. Considering Spoofing attacks, Bernoulli random variables are used for statistics [28]. The output measurement value under Spoofing attacks is $\bar{y}=\alpha (t)(\zeta (t)-y(t))+y(t)$, combined with Spoofing attacks and DoS attacks, interconnected power LFC model actual output under mixed attack is shown in Eq (25).


$$y(t)=\alpha (t)\zeta (t)-(1-\alpha (t))(e_{\hat{x}}(i_{k}h)+Cx(t-\eta (t))),t\in \Omega_{l}$$ (25)

**Fig 4:**
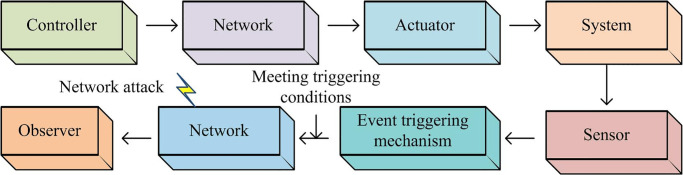
Schematic diagram of interconnected power network under mixed attack.

The schematic diagram of the interconnected power network under mixed attacks is shown in Fig 4.

From this, the expression of the interconnected power LFC model under mixed attacks can be obtained as shown in Eq (26).


$$\left\{\begin{array}{l} \dot{x}(t)=F\omega (t)+Bu(t)+Ax(t)\\ y(t)=\alpha (t)\zeta (t)-(1-\alpha (t))C(x(t-\eta (t))-e_{\hat{x}}(i_{k}h)),t\in \Omega_{l} \end{array}\right.$$ (26)

Similarly, based on the observer function, the expression of the sliding mode surface is obtained in Eq (27).


$$s(t)=B^{T}X[\hat{x}(t)-\int_{0}^{t}\left(A+BK\right)\hat{x}(\theta )d\theta ]$$ (27)

A controller is designed as shown in Eq (27) to converge the system onto the desired surface, as shown in Eq (28).


$$u(t)=-\beta s(t)+K\hat{x}(t)-\Psi (t)\cdot \text{sgn}(s(t))$$ (28)

In Eq (28), *β* represents a positive definite constant and sgn(⋅) represents a sign function. The expression Ψ(*t*) is shown in Eq (29).


$$\Psi (t)=\|{\left(B^{T}XB\right)}^{-1}\|[\|B^{T}XL\zeta (t)\|+2\|B^{T}XLe_{\hat{x}}(i_{k}h)\|+2\|B^{T}XLC\varpi (t-\eta (t))\|]$$ (29)

**Fig 5:**
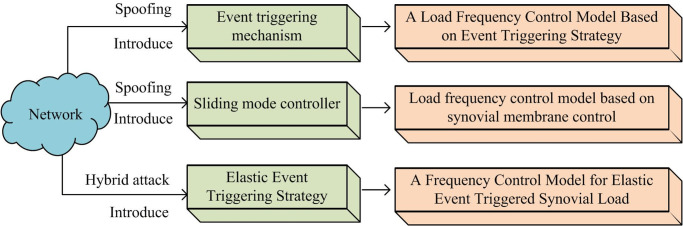
Flow chart of model construction.

According to the designed sliding mode LFC scheme based on elastic event-triggered under mixed attacks, it ensures that the power system can reduce the burden of network bandwidth under certain control performance. At the same time, it can also maintain stable performance of the interconnected power system under mixed attacks. The model construction process diagram for this study is shown in Fig 5.

## 4. Simulation experiments and analysis

To verify model effectiveness, this chapter is divided into three parts for simulation testing, analyzing the application effects of different control strategies under different spoofing attacks. The first part conducts simulation testing on the load control model based on an event-triggered strategy and analyzes its effectiveness under spoofing attacks. In the second part, the limitations of the method outlined in the first part become apparent when significant changes occur in the control system input. To address this, a new approach incorporating SMC is introduced, and simulation tests are carried out to analyze the LFC model, validating the efficacy of SMC and event-triggered strategies. Subsequently, in the third part, the method from the second part is adjusted to account for multiple attacks, leading to the introduction of an elastic triggering strategy for simulating and analyzing the sliding mode LFC model, thereby confirming the effectiveness of elastic-event-triggered SMC.

### 4.1. LFC model simulation testing and analysis based on event-triggered strategy

This study analyzed a three-region interconnected power system. The experiment was simulated on MATLAB software for effectiveness verification of the designed LFC model based on an event-triggered strategy. The parameter settings are shown in Table 1.

**Table 1:** Parameter setting table.

Parameter	Setting values
*A*	$\left[\begin{array}{cc} 0.94 & -0.8\\ -0.14 & 0.3 \end{array}\right]$
*G*	$\left[\begin{array}{c} -3\\ -0.6 \end{array}\right]$
*J*	$\left[\begin{array}{c} 0.08\\ -0.04 \end{array}\right]$
*F*	$\left[\begin{array}{cc} 0.002 & 0.03\\ 0.03 & -0.03 \end{array}\right]$
Sampling period *h*	0.01
Threshold parameter *ρ*	0.4
*H*_∞_’s anti-interference level *γ*	1.44
Attack probability	0.4
Time window	3

**Fig 6:**
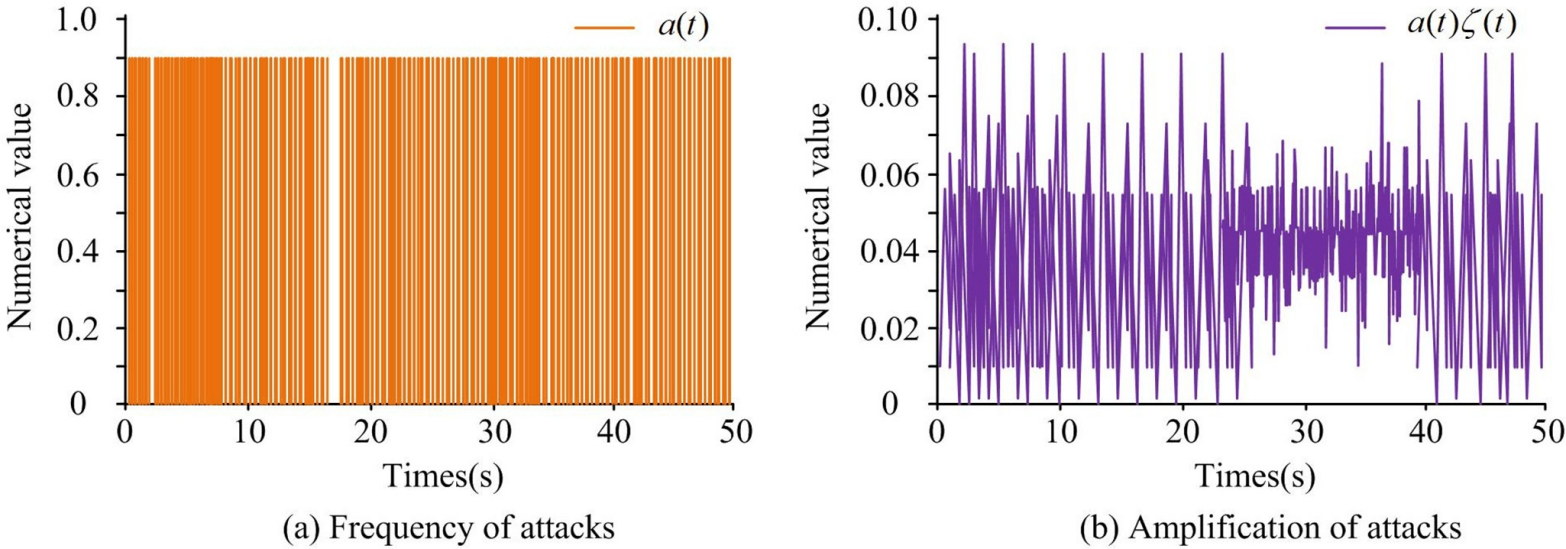
Frequency and amplitude of deceptive random attacks.

In Table 1, different parameter values are set, with a sampling period of 0.01s. Due to the significant impact of the sampling period *h* on the frequency stability, if the sampling period was too large, it can cause the loss of control signal packets. Therefore, to lighten the communication network load, it was more appropriate to set *h* to 0.01. The PI-based controller had a gain of 0.1 and an integral gain of 0.1, *T*_12_ = 0.2(*pu*/*rad*), *T*_13_ = 0.12(*pu*/*rad*), *T*_23_ = 0.25(*pu*/*rad*). The sample size was 100. The frequency and amplitude of deceptive random attacks are shown in Fig 6.

**Fig 7:**
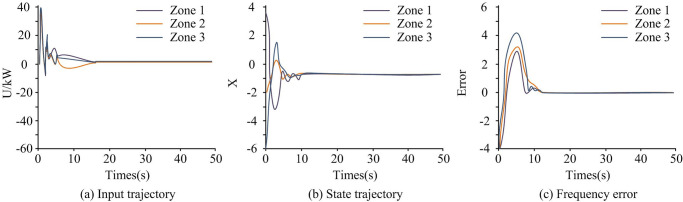
System test results.

To verify control performance of power network systems under deceptive random attacks, control input and state trajectory, and system frequency error were tested. The control input trajectory was usually used to represent the control of generator output power and load control. State trajectories were used to represent the dynamic changes of various components in the power system. The results are shown in Fig 7.

**Fig 8:**
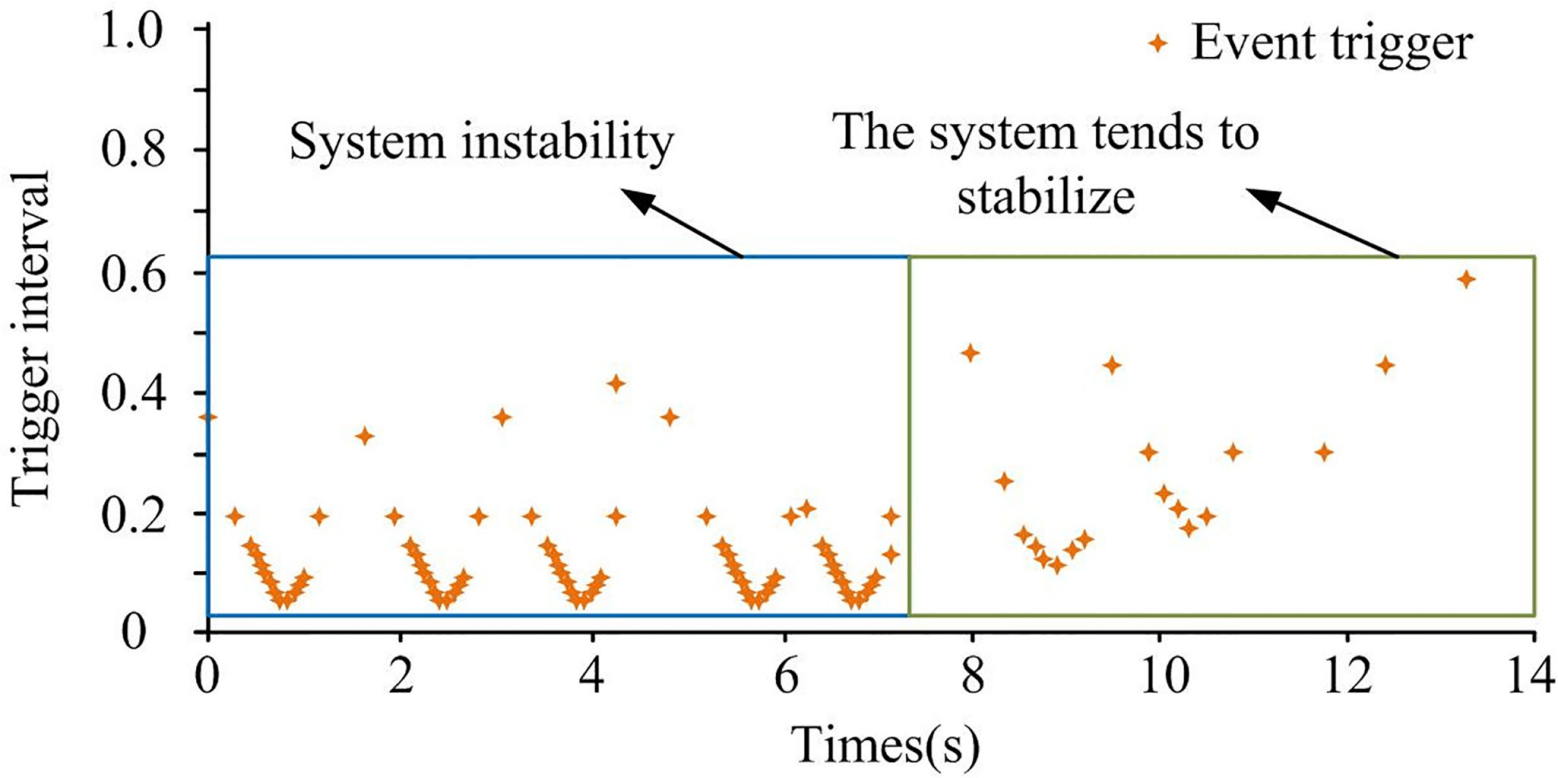
Trigger interval diagram.

Fig 7(a) shows the control input trajectory of the system, Fig 7(b) shows the state trajectory of the system, and Fig 7(c) shows the frequency error of the system. From Fig 7(a), the system voltage input was fluctuated in the early stages of being subjected to deceptive random attacks in the three regions. In the first 5 seconds, the input fluctuation of the system was strong, with a maximum of 40kW. During the 5s to 15s phase, the input fluctuations of the system gradually flattened out. At the 15th second, the voltage in the three regions tended to stabilize. At this point, the voltage input was small, close to 0kW. Therefore, the system voltage was also in a stable state. In Fig 7(b), during the first 10 seconds, the voltage state trajectories of the three regions fluctuated significantly. Over time, the voltage state trajectory of the system tended to stabilize after 10 seconds. In Fig 7(c), the frequency deviation of the interconnected power system composed of three regions can be stabilized at 12 seconds. At this point, the system frequency error was relatively small, and the corresponding error value was 0. When the time was 5 seconds, the system frequency error in the three regions was the largest, especially in region three, which corresponded to an error value of 4.13. Between 5s and 12s, the system frequency error in the three regions first rapidly decreased, then increased in small ranges, and finally stabilized. Overall, the frequency deviation of the controlled interconnected power system can be stabilized at around 15 seconds, indicating the effectiveness of the proposed method, the control effect of the research method was good. When subjected to deceptive random attacks, the triggering interval of the event-triggered strategy is shown in Fig 8.

**Fig 9:**
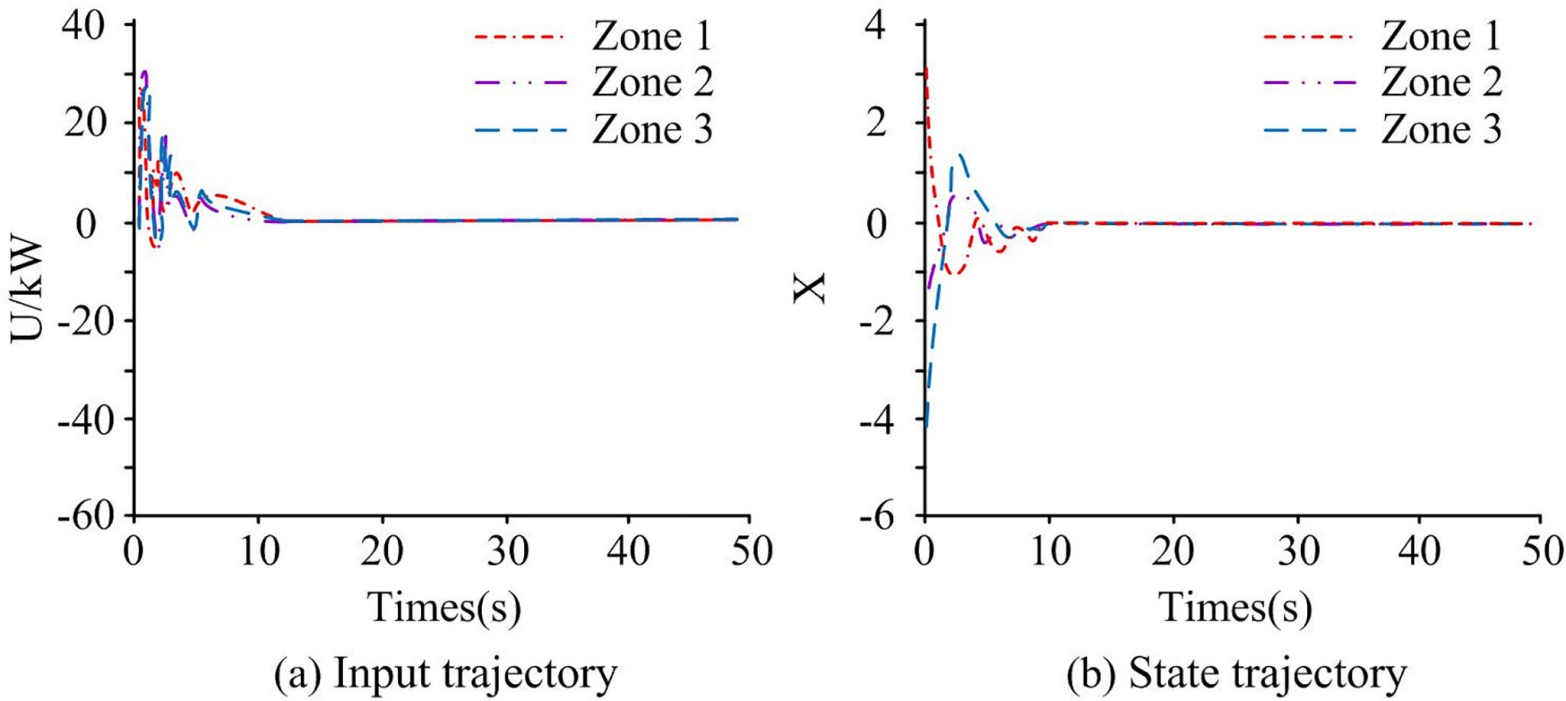
System test results.

Combining Figs 8 and 7, in the early stages of the power system being attacked, the triggering interval of the event-triggered strategy was relatively small. The event-triggered strategy at this time reduced the interval between the time of information transmission and the time of adjacent information transmission, enabling the system to recover to a stable state as soon as possible. As the attack time increased, the system gradually stabilized, and the time trigger interval was relatively large. In Fig 8, it avoided a large amount of unnecessary continuous information exchange, effectively saving limited network communication resources. Therefore, it indicated that when facing deceptive random attacks, the introduced event-triggered strategy enabled the control of the system to achieve a good effect. Using the same method, the control input and state trajectory of the system when facing false data injection attacks were analyzed, as shown in Fig 9.

Compared with Fig 7(a), the fluctuation amplitude of the curve in Fig 9(a) slowed down to a certain extent, and the voltage in the three regions stabilized faster. During the initial stage of false data injection attacks, the voltage input of the system in three regions was in a fluctuating state. In the first 2 seconds, the input of the system fluctuates strongly, reaching a maximum of 31kW. During the 2s to 12s phase, the input fluctuations of the system gradually stabilized. At the 12th second, the voltage in the three regions tended to stabilize. At this point, the voltage input was relatively small, approaching 0kW. Compared with Fig 7(b), the fluctuation amplitude of the curve in Fig 9(b) slowed down to a certain extent, and the voltage states in the three regions stabilized faster. During the first 10 seconds, the voltage state trajectories of the three regions fluctuated significantly. As time went on, the voltage state trajectory of the system tended to stabilize after 10 seconds. The control effect of the research method was good.

### 4.2. LFC model simulation testing and analysis based on smc

**Fig 10:**
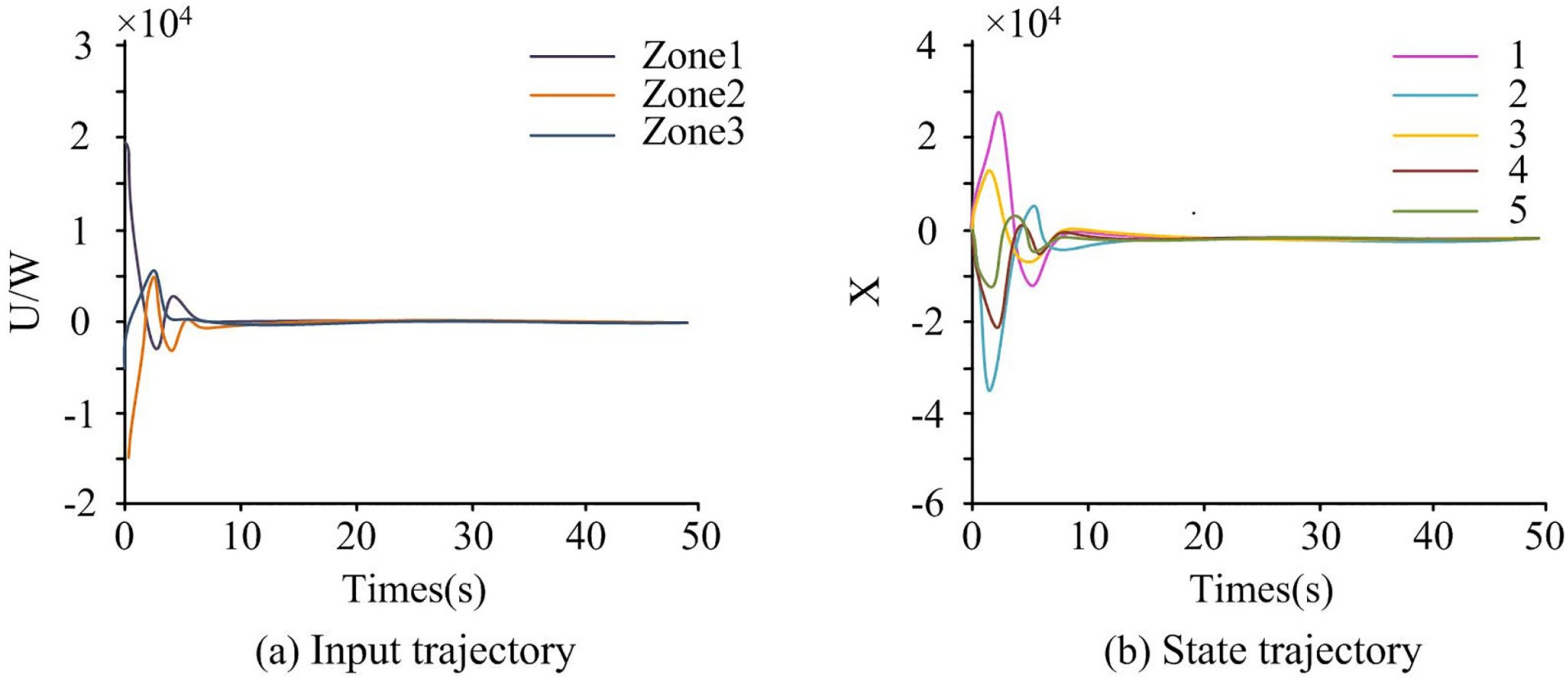
System control input trajectory and state trajectory.

To verify the effectiveness of SMC, simulation tests were conducted in a three-zone power system. The sampling period *h* was also set to 0.01s, threshold parameter *ρ* to 0.4, and *η*(*t*) to 0.1. The Longberg observer gain matrix *L* was represented as *L* = *X*^−1^*Y*. *x*(*t*) was set to 1–5, and the corresponded $\hat{x}(t)$ was set to 1–5. Fig 10 shows the system control input trajectory and state trajectory.

**Fig 11:**
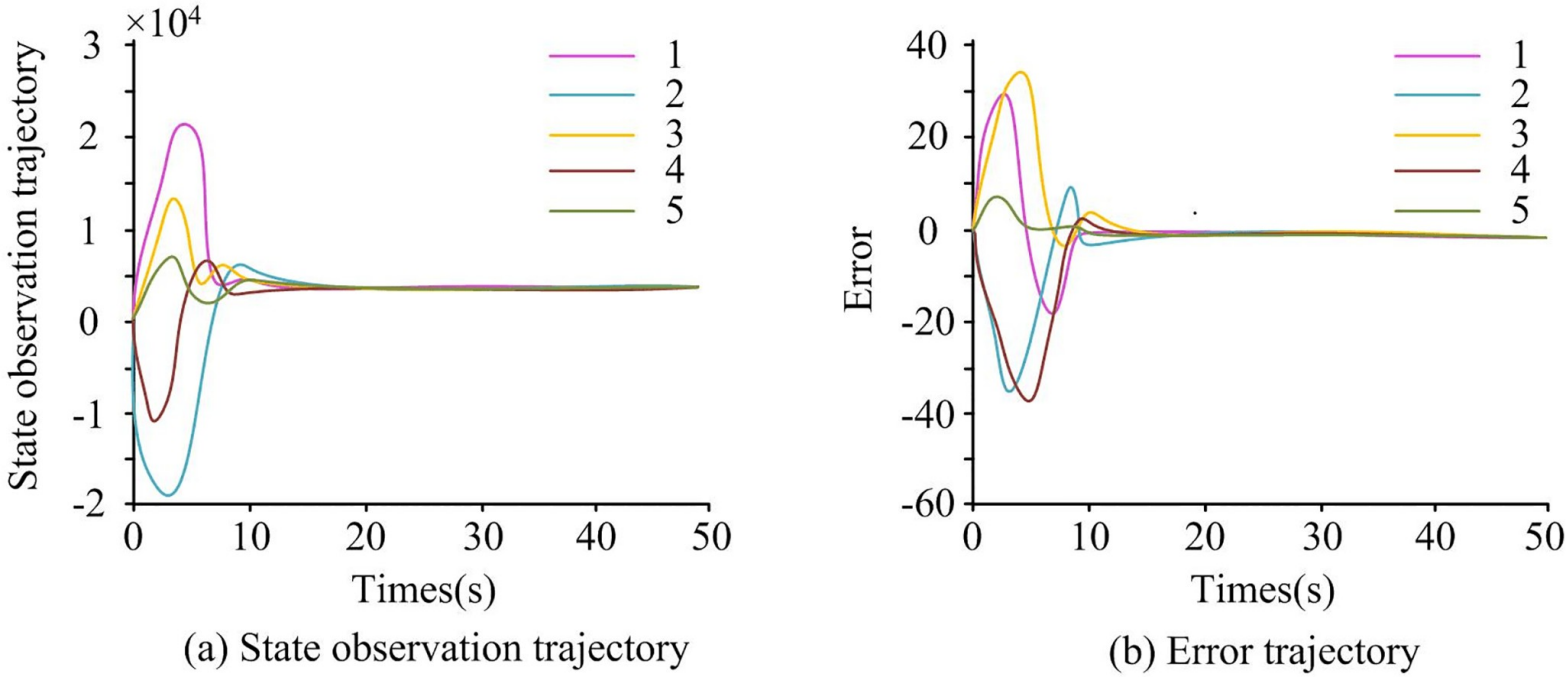
State observation trajectory and system error.

Fig 10(a) represents the system control input trajectory, and Fig 10(b) represents the system state trajectory. In this stage, there were differences in the voltage input situation among the three regions. The voltage input in region 1 first rapidly decreased, then increased in a small range, and then gradually decreased, finally tending towards 0W. When the time was 2.5 seconds, the maximum voltage input value for Zone 3 was 0.57 * 104W, followed by Zone 2, which was 0.50 * 104W. From Fig 10(a), the lines fluctuated significantly in the first 10 seconds, indicating that the system was under attack and was in a state of adjustment. In Fig 10(b), the system state trajectory fluctuated significantly in the first 10 seconds. This indicates that the system was currently under a spoofing attack, causing a change in the system’s load. But after 10 seconds, the system was in a stable state. This indicates that SMC can enable the system to undergo adaptive adjustment and achieve stable operation. Overall, the control effect of the research method was good. The state observation trajectory and system error of the power system are shown in Fig 11.

The numbers in Fig 11 represent $\hat{x}(t)$. In Fig 11(a), the large fluctuations in the state trajectory indicate that the system was under the interference influence of spoofing attacks, and the dynamic performance of the system had not yet fully recovered and stabilized. As time passed, at the 10th second, the system gradually adapted and adjusted, and the state trajectory tended to stabilize and approach the expected operating state. In Fig 11(b), the control input trajectory fluctuated significantly in the first 10 seconds, and the system was under Spoofing attacks, resulting in a certain degree of error or instability. As time went on, at the 10th second, the system gradually stabilized by adjusting the control input, and the error gradually decreased and approached the expected control goal.

**Fig 12:**
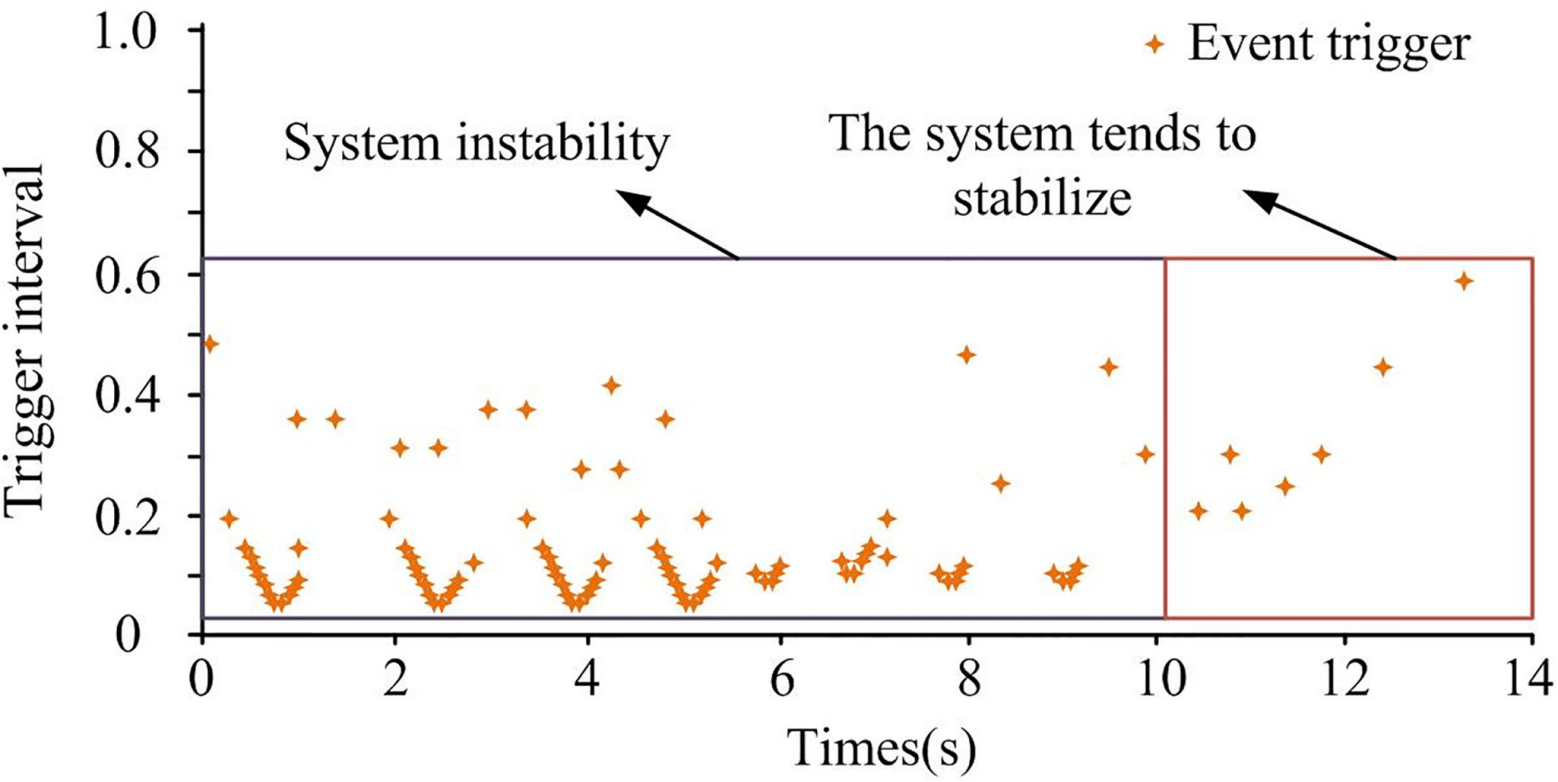
Trigger interval diagram.

In Fig 12, the triggering time interval of the event-triggered strategy was initially small, and gradually increased later, usually indicating that the system was gradually adjusting and adapting. Specifically, the small triggering time interval in the initial stage was due to the system being in a state of attack and requiring quick response and emergency control. As time went by, the system gradually adjusted and adapted, and the triggering time interval gradually increased to reduce the frequency of calculation and control and reduce system load. Therefore, it was proven that event-triggered strategies can effectively reduce network transmission load.

### 4.3. Elastic event-triggered sliding mode LFC model simulation testing and analysis under various attacks

**Fig 13:**
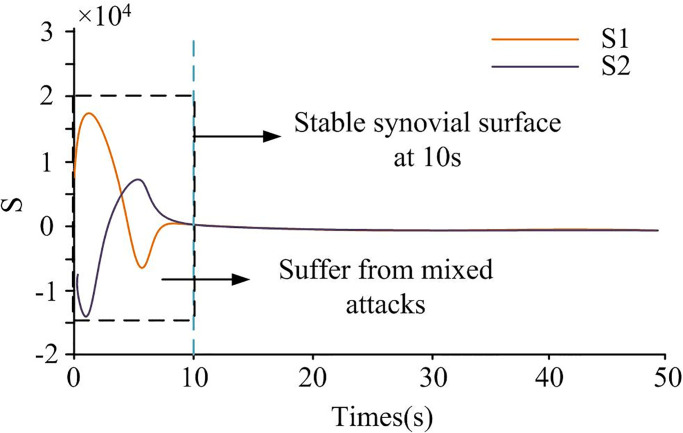
Sliding mode surface trajectory diagram.

To verify the effectiveness of the LFC model triggered by elastic events in SMC under mixed attacks, the experiment was conducted in an interconnected power system between two regions for simulation. The three-region interconnected power system consisted of three regions, while the two-region interconnected power system consisted of two regions, with different numbers of region sincluded. Compared to the three-region interconnected power system, the sampling period of the two-region interconnected power system was longer, with the sampling period of the previous system being 0.04 seconds less than the latter. Two-region interconnected power systems suffered from multiple attacks, while three-region interconnected power systems suffered from a single attack. The flexible event-triggered scheme was designed in the system, which had flexibility for DoS control. Therefore, at this point, *φ*(*i*_*k*_*h*) was set to 1, the probability of spoofing attacks occurring to 0.2, sampling period *h* to 0.05s, and the threshold parameter *ρ* to 0.4. *x*(*t*) was set to 1–5, the corresponded $\hat{x}(t)$ was set to 1–5. The sliding mode surface constructed by this research institute is shown in Fig 13.

**Fig 14:**
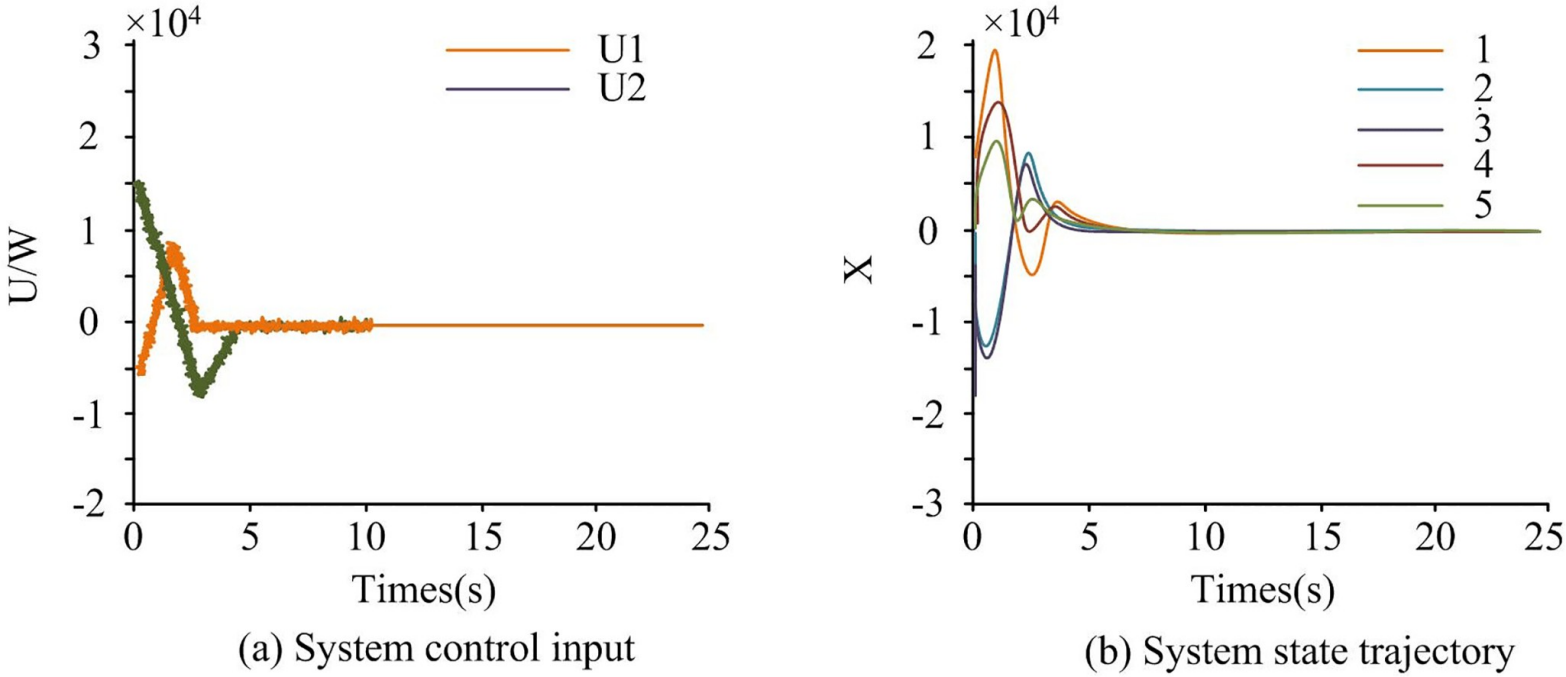
Control input and system state trajectory.

In Fig 13, S1 and S2 represent the interconnected power system between two regions. In Fig 13, during the first 10 seconds, the sliding mode surface was fluctuating due to mixed attacks on the system. At the 10th second, the sliding mode surface was in a stable state. To verify whether the control model can converge the system onto the desired surface, the control input and system state trajectory were simulated. Results are shown in Fig 14.

**Fig 15:**
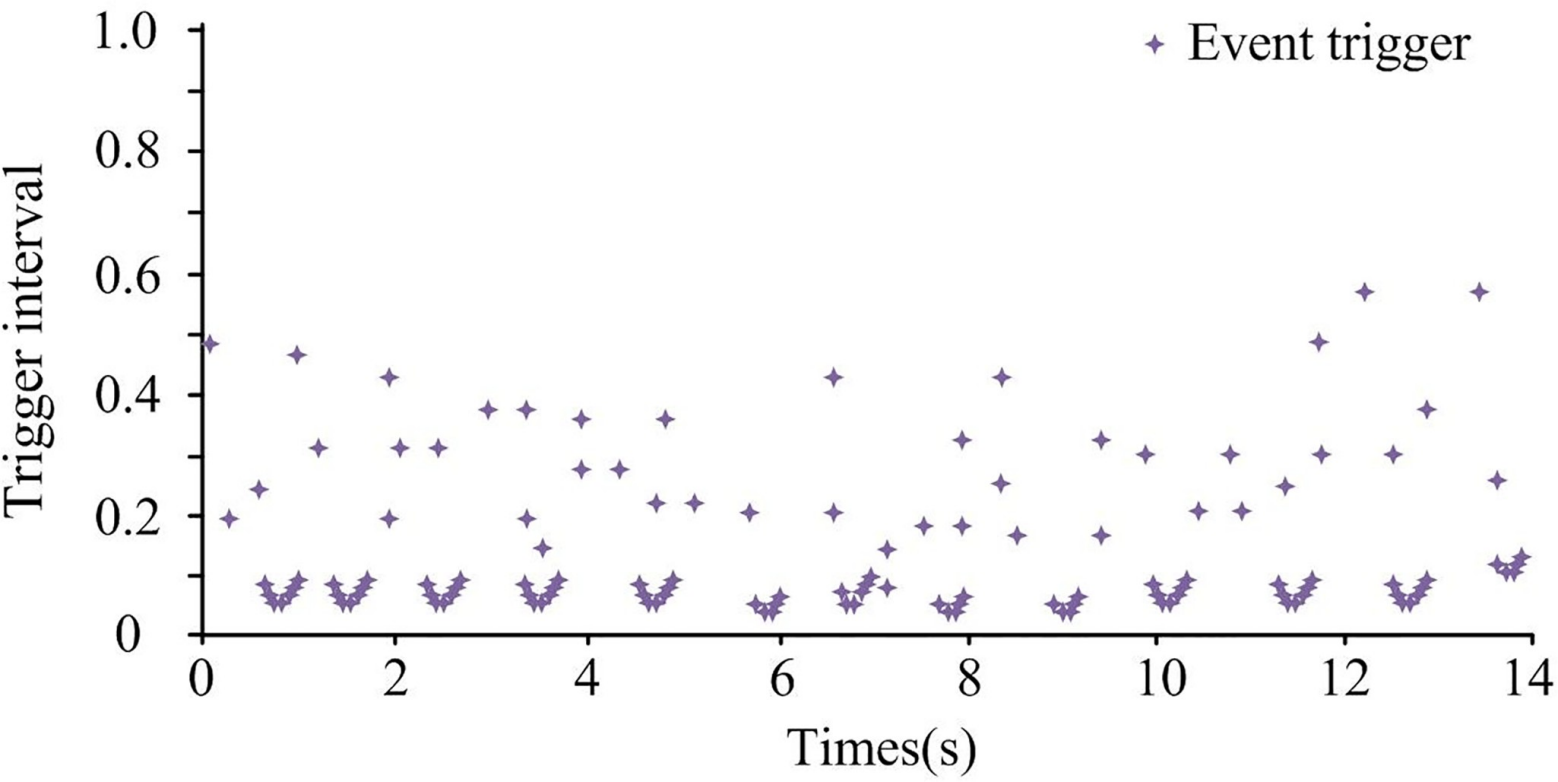
Trigger interval of elastic event-triggered strategy under mixed attacks.

U1 and U2 in Fig 14 represent the voltage of the two regions, with numbers 1 to 5 indicating $\hat{x}(t)$. In Fig 14(a), as time passed, the voltage in Region 1 first rapidly decreased, then increased, and then stabilized, remaining at 0W. The voltage in Region 2 first rapidly increased, then decreased, and then stabilized, remaining at 0W. The two regions began to stabilize at 12s, with a faster rate of stabilization. In Fig 14(b), the trajectories of different system states exhibited varying degrees of fluctuation before 7 seconds and tended to stabilize around 7 seconds. In Fig 14, the constructed control model can converge the system to the sliding mode surface. Therefore, the validity of Eq (27) has been proven. The triggering interval of the elastic event-triggered strategy under mixed attacks is shown in Fig 15.

From Fig 15, the elastic event-triggered strategy ensured that the system gradually adjusted under mixed attack conditions. The later interval gradually increased, reducing the frequency of system control and reducing system load. Therefore, Fig 15 also proves that the event-triggered strategy can effectively reduce network transmission load. The control effectiveness of the research method was analyzed and compared with the fuzzy logic method in reference [8]. The multi-region power system was the same as above, and the time when the system started to stabilize under the two methods is shown in Table 2.

**Table 2:** Comparison results of two methods.

Time when the system starts to stabilize (s)	Research method	The fuzzy logic method
System frequency error	10	15
System state trajectory	7	13
System control input	10.6	17

In Table 2, from the time when the system began to stabilize, the performance of the research method was better. In the system frequency error, it stabilized faster with a corresponding time of 10 seconds, which was 5 seconds smaller than the fuzzy logic method. In the system control input trajectory, compared to the fuzzy logic method, the voltage of the research method stabilizes faster, with a starting time of 10.6 seconds, which is 6.4 seconds less than the fuzzy logic method. In terms of system state trajectory, the research method and the fuzzy logic method have a voltage state stabilization time of 7 seconds and 13 seconds, respectively. The control effect of the research party was good. Overall, the research validated the control methods designed through a three-region interconnected power system and a two-region interconnected power system, and these experiments were successfully conducted, indicating the practicality of the methods. From the results of system control inputs, state trajectories, and other related factors, it has been confirmed that the system can quickly stabilize under deceptive attacks, which means that the research and design method is effective and feasible.

## Conclusion

Due to the vulnerability of interconnected power network systems to attacks, to maintain power system stability and reduce the impact of load frequency and delay. This study is based on event-triggered and constructs a LFC model based on event-triggered strategy under spoofing attacks. It also introduces a sliding mode controller and constructs an LFC model based on SMC. Based on DoS attacks and spoofing attacks, a frequency control model for elastic event-triggered sliding mode load is constructed. The simulation experiment results showed that when the three regions were subjected to deceptive random attacks, the input fluctuation of the system was strong in the first 5 seconds, with a maximum of 40kW. During the 5s to 15s phase, the input fluctuations of the system gradually flattened out. At the 15th second, the voltage in the three regions tended to stabilize. Meanwhile, three regions combined interconnected power system frequency deviation can be stabilized at 12 seconds. The triggering time interval of the event-triggered strategy proved that the proposed strategy reduced network transmission load. The system state trajectory fluctuated significantly in the first 10 seconds. This indicated that the system was currently under a spoofing attack, causing a change in the system’s load. But after 10 seconds, the system was in a stable state. This indicated that SMC enabled the system to undergo adaptive adjustment and achieve stable operation of the power system. Under mixed attacks, the constructed control model converged the system to the sliding mode surface within a limited amount of time. Moreover, the elastic event-triggered strategy reduces the frequency of system control and reduces system load. There are still some shortcomings in the research, as it does not consider the potential packet loss caused by network attacks. When this problem occurs, it will affect the stability of the system and may result in incomplete information received by the control system, leading to the inaccurate response of the control system based on incomplete or outdated data. Data packet loss may cause system operators or automatic control systems to lack sufficient information to maintain frequency and voltage stability, leading to fluctuations. This may have a certain impact on the control effect of the method and reduce its applicability. Among them, if data packet loss leads to inaccurate or incomplete system monitoring data, it may affect the accurate perception of the power system state by the sliding membrane control model. Therefore, the adaptive adjustment ability of the synovial control model may be disturbed, thereby affecting its stability and performance. In future research, these factors’ impact on the power network can be further understood.

## Supporting information

S1 File Justification and unique contribution. (DOCX)
