# Supplementary material for: Security load frequency control model of interconnected power system based on deception attack
Source: PLoS One. 2024 Feb 29;19(2):e0298889. doi: 10.1371/journal.pone.0298889 (PMC10903862; doi:10.1371/journal.pone.0298889)
Supplement: S1 File — (DOCX) [file pone.0298889.s001.docx]

In order to improve resource utilization and avoid hacker intrusion, this study constructs a load frequency control model based on event triggered and spoofing attack strategies under spoofing attacks. And introduce a sliding film controller to construct a load frequency control model based on sliding film control. Based on various attack methods, a Runberg observer is introduced to construct a frequency control model for elastic event triggered synovial load.

Load frequency control is achieved by monitoring changes in system frequency and adjusting the output of the generator to maintain the system frequency at a predetermined value. By adjusting the power flow through a sliding film controller, the node voltage in the system can be maintained stable and able to cope with system changes and fault situations. Therefore, this study constructs an event triggered strategy based load frequency control model under deception attacks based on event triggered strategies. Introduce a sliding film controller and construct a load frequency control model based on sliding film control. And construct an elastic event triggered synovial load frequency control model under deception attacks and denial of service attacks.

Load frequency control and sliding film control strategies can significantly improve the stability and robustness of power systems. However, existing research has limited the fusion of the two methods. Moreover, there is a lack of research on the sliding membrane load control of interconnected power systems under attack. Therefore, this study constructs an event triggered strategy based load frequency control model under deception attacks based on event triggered strategies. And introduce a sliding film controller and a Longberg observer to construct a load frequency control model based on sliding film control. And construct an elastic event triggered synovial load frequency control model under deception attacks and denial of service attacks.
